# Pathological macromolecular crystallographic data affected by twinning, partial-disorder and exhibiting multiple lattices for testing of data processing and refinement tools

**DOI:** 10.1038/s41598-018-32962-6

**Published:** 2018-10-05

**Authors:** Ivan Campeotto, Andrey Lebedev, Antoine M. M. Schreurs, Loes M. J. Kroon-Batenburg, Edward Lowe, Simon E. V. Phillips, Garib N. Murshudov, Arwen R. Pearson

**Affiliations:** 1 Astbury Centre for Structural Molecular Biology, University of Leeds, Leeds, LS2 9JT UK; 2 Biochemistry Department, Oxford University, South Parks Road, Oxford, OX1 1HY UK; 3 Leicester Institute of Structural and Chemical Biology, University of Leicester, Lancaster Road, Leicester, LE1 7RH UK; 4 Research Complex at Harwell (RCaH), Rutherford Appleton Laboratory, Harwell Science and Innovation Campus, Didcot, Oxford OX11 OFA UK; 5 Department of Crystal and Structural Chemistry, Bijvoet Center for Biomolecular Research Utrecht University, Padualaan 8, 3584 CH Utrecht, The Netherlands; 6 Structural Studies Division, MRC-LMB, Francis Crick Avenue, Cambridge, CB2 0QH UK; 7 Hamburg Centre for Ultrafast Imaging, Institute of Nanostructure and Solid State Physics, Universität Hamburg, CFEL, Luruper Chaussee 149, 22761 Hamburg, Germany

## Abstract

Twinning is a crystal growth anomaly, which has posed a challenge in macromolecular crystallography (MX) since the earliest days. Many approaches have been used to treat twinned data in order to extract structural information. However, in most cases it is usually simpler to rescreen for new crystallization conditions that yield an untwinned crystal form or, if possible, collect data from non-twinned parts of the crystal. Here, we report 11 structures of engineered variants of the *E. coli* enzyme N-acetyl-neuraminic lyase which, despite twinning and incommensurate modulation, have been successfully indexed, solved and deposited. These structures span a resolution range of 1.45–2.30 Å, which is unusually high for datasets presenting such lattice disorders in MX and therefore these data provide an excellent test set for improving and challenging MX data processing programs.

## Introduction

Twinning is a crystal growth anomaly or lattice disorder in which the crystal is composed of separate domains of differing orientations^1^. Twinning has posed a challenge in macromolecular crystallography since the earliest days^2,3^ and multiple computational approaches have been developed in order to treat twinned data in order to extract structural information. Several exhaustive reviews are available that discuss twinning and the methods to address it in detail^1,4–7^ nevertheless, for clarity, we give here a brief description of this phenomenon. Twinning is characterised by the twin law (a set symmetry operators, which relate the different orientations of the domains); and the twin fractions, αι, that characterise the relative volumes of the twinning domains. There are several types of twinning: merohedral twinning (when the twin operators are a subset of the exact rotational symmetry of the lattice); pseudo-merohedral twinning (when the twin operators approximate the rotational symmetry of the lattice); and non-merohedral twinning or epitaxial twinning (when the twin operators have the rotational symmetry of a sublattice in three or fewer dimensions). In this paper we present examples of pseudo-merohedral twinning.

When there are two twin domains, and the twin operator is a 2-fold rotation, the twinning is called hemihedral twinning. When the twin domains are sufficiently large, the diffracted waves from these domains do not interfere (or interference is negligible, depending on the coherence radius of the beam and twin domain sizes) and the observed intensities are simply the weighted sum of the intensities from each of the individual domains^8^. If the twin fraction α approaches 0.5 the diffraction pattern acquires an additional symmetry, imposed by the twinning operator, which may lead to erroneous indexing in a higher symmetry space group. If the twin fraction equals 0.5, the crystal is perfectly twinned and the intensity measurements cannot be deconvoluted. If the twin fraction is <0.5 it is possible to deconvolute the data in order to recover the untwinned intensities^1^. However, errors in the deconvoluted intensities increase proportionally and can become a very large fraction of the intensities as the twin fraction approaches 0.5. Twinning can thus hamper crystal structure determination at all stages, from indexing, to data reduction, phase determination and refinement.

Since the intensities of twin related reflections are correlated, twinning reduces the information content of the data. In the limit case of perfect merohedral twinning that reduction is equivalent to a reduction in the resolution limit by a factor of 1.26. An additional complication is that the statistical properties of the data from twinned and untwinned crystals are different and therefore overall statistics describing model quality such as the R_factor_/R_free_ must be interpreted with extra care^9^. In particular, the gap between R_factor_ and R_free_ values as well as their individual values needs to be monitored during refinement. If refinement using the twin option leads to an increase of the gap between R_factor_ and R_free_, this indicates a serious problem with the refinement protocol and data handling.

Another type of deviation from perfect periodicity in a crystal, is crystal modulation, in which the content of asymmetric unit is not perfectly replicated by the lattice operations and which can occur with a period commensurate or incommensurate with the lattice periodicity. As result of crystal modulation, primary Bragg reflections are flanked by off-lattice satellite reflections^10^. The direction and magnitude of such satellite reflections is described by an additional vector **q**, which needs to be added to the reciprocal space vector **H** to define a 4-dimensional reciprocal space vector. Although incommensurate crystals have been reported rarely in macromolecular protein crystallography^11,12^, the EVAL software suite can index and process such data^10,13^, and in silico simulations of modulated structure have been performed^14^.

In this report we present 11 diffraction data sets, in multiple space groups, from the *E. coli* enzyme *N*-acetyl-neuraminic acid lyase (NAL), which present twin lattices and incommensurate modulation. NAL is a tetramer in solution, that crystallises in low salt conditions^15^ to give four different crystal forms, three in space group *P*2_1_ and one in *P*2_1_2_1_2_1_^15–17^. Interestingly, the three crystal forms in space group P2_1_ were not related to each other, two of them were twinned and shared the same twinning operator, which made the monoclinic cells a pseudo-orthorombic cell.

Two of the crystal forms are reported here for the first time, and some were pseudo-merohedrally twinned with the additional complication of incommensurate modulation. Although they could all be solved by molecular replacement, they could not be refined satisfactorily using standard protocols. However, with improvements in REFMAC5, one of the software packages for macromolecular structure refinement available from ccp4 suite 7.0^18^, with direct contribution from the presented test cases, we were able to refine models satisfactorily against all 11 datasets. Due to the varied diffraction data pathologies (pseudo-merohedral twinning with α up to 0.497 as well as crystal modulation) we believe these data form a useful test set for the development of macromolecular crystallographic data processing and structure refinement software and therefore we made them available to the community through the public repository Zenodo (public links in Data Records).

## Results and Discussion

### Data processing

NAL crystallised in four different crystal forms from the same crystallisation conditions, and in the same drops (100 mM Tris-HCl pH 8.0–8.2, 200 mM Na acetate, 18–22% w/v PEG3350). It was not possible to discriminate between the four crystal forms of NAL solely by inspection of the crystal morphology (Fig. S1). The most commonly obtained crystal form (I) belonged to space group *P*2_1_ with unit cell parameters a = 55 Å, b = 142 Å, c = 84 Å, β = 109° (decimals are omitted due to variability between datasets); followed by crystal forms II (a = 84 Å, b = 95 Å, c = 91 Å, α = 90°, β = 116°, γ = 90°) and III (a = 78 Å, b = 108 Å, c = 148 Å, α = 90°, β = 116°, γ = 90°), both in space group *P*2_1_, and crystal form IV (a = 78 Å, b = 116 Å, c = 84 Å, α = β = γ = 90°) in space group *P*2_1_2_1_2_1_ (Table S1).

**Figure 1:**
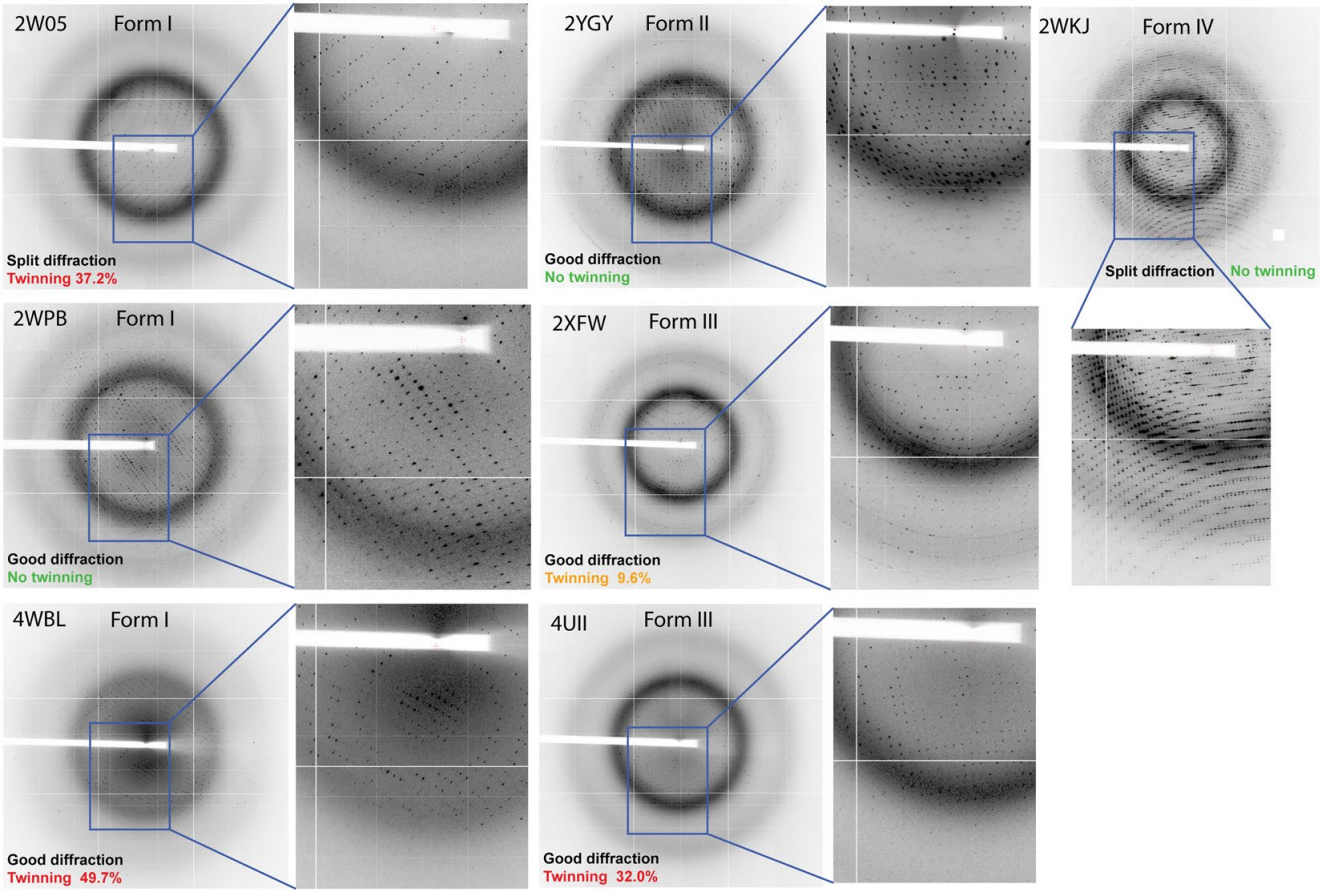
Diffraction pattern typologies observed for the crystal forms I, II, III and IV of NAL.

The diffraction patterns occasionally showed spot splitting in all four crystal forms of NAL and it was not possible to predict the successful indexing and scaling outcome based on the observed diffraction quality alone (Fig. 1). The main and satellite reflections are clearly distinct and the main lattice could be indexed separately while satellite reflections were ignored by MOSFLM^19^ (i.e. PDB 2WNN, 2WPB & 2WKJ, Fig. 1).

Closer inspection of the diffraction pattern of four of the seven datasets in crystal form I with DIALS viewer^20^ revealed two lattices but also some extra reflections, which did not belong to either lattices (Fig. S2). MOSLFLM successfully indexed the main lattice in all cases (Table 1), but we decided to further investigate whether these extra reflections in the diffraction pattern could be caused by crystal modulation, as they appeared to be occurring in a periodic manner.

**Table 1:** Improvement of refinement statistics upon applying the twin option in REFMAC.

Datasets	Res	Crystal form and cell parameters	Obliquity* (ω)	Twinning fraction*	Twin on	Twin off	PDB code	Diamond Station
Wild type apo	2.20 Å	P2_1_ crystal form I a = 54.8 b = 142.2 c = 84.2 α = 90.00 β = 108.97 γ = 90.00	0.019	0.372	R_factor_ = 0.200 R_free_ = 0.267	R_factor_ = 0.251 R_free_ = 0.319	2WO5	I02
Wild type pyruvate complex	1.65 Å	P2_1_ crystal form I a = 54.7 b = 142.5 c = 83.6 α = 90.00 β = 109.16 γ = 90.00	0.070	0.334	R_factor_ = 0.201 R_freev_ = 0.245	R_factor_ = 0.256 R_freev_ = 0.293	2WNN	I03
E192N apo	1.80 Å	P2_1_ crystal form I a = 54.6 b = 142.8 c = 84.3 α = 90.00 β = 108.8 γ = 90.00	0.130	0.463	R_factor_ = 0.195 R_free_ = 0.244	R_factor_ = 0.272 R_free_ = 0.320	2WNQ	I04
E192N pyruvate complex	1.80 Å	P2_1_ crystal form I a = 56.9 b = 143.0 c = 83.9 α = 90.00 β = 109.8 γ = 90.00	0.000	**—**	R_factor_ = 0.178 R_free_ = 0.209	R_factor_ = 0.187 R_free_ = 0.223	2WNZ	I02
E192N + pyruvate + THB**	2.05 Å	P2_1_ crystal form I a = 57.0 b = 143.7 c = 84.3 α = 90.00 β = 109.9 γ = 90.00	0.130	**—**	R_factor_ = 0.192 R_free_ = 0.238	R_factor_ = 0.191 R_free_ = 0.242	2WPB	I03
Y137A pyruvate complex	1.80 Å	P2_1_ crystal form I a = 54.7 b = 142.2 c = 83.6 α = 90.0 β = 109.0 γ = 90.0	0.119	0.149	R_factor_ = 0.287 R_free_ = 0.331	R_factor_ = 0.296 R_free_ = 0.357	n./a.***	I04
Y137A pyruvate, ManNAc and Neu5Ac complex	2.00 Å	P2_1_ crystal form I a = 56.1 b = 143.5 c = 83.6 α = 90.0 β = 109.6 γ = 90.0	0.094	0.497	R_factor_ = 0.183 R_free_ = 0.236	R_factor_ = 0.265 R_free_ = 0.321	4BWL	I02
Wild type apo	1.90 Å	P2_1_ crystal form II a = 84.3 b = 95.9 c = 91.4 α = 90.00 β = 115.33 γ = 90.00	2.10	**—**	R_factor_ = 0.198 R_free = _0.226	R_factor_ = 0.197 R_free = _0.225	2YGY	I02
E192N/Y137F pyruvate complex	1.80 Å	P2_1_ crystal form III a = 78.0 b = 116.7 c = 83.7 α = 90.0 β = 118.06 γ = 90.00	0.290	0.328	R_factor_ = 0.156 R_free_ = 0.183	R_factor_ = 0.206 R_free_ = 0.228	2YGZ	I02
E192N + pyruvate complex	1.85 Å	P2_1_ crystal form III a = 78.1 b = 116.5 c = 83.7 α = 90.00 β = 116.5 γ = 90.00	0.150	0.096	R_factor_ = 0.165 R_free_ = 0.186	R_factor_ = 0.174 R_free_ = 0.193	2XFW	I02
E192N + pyruvate	1.45 Å	P2_1_2_1_2_1_ crystal form IV a = 78.3 b = 108 c = 148.3 α = β = γ = 90.00	0.000	**—**	R_factor_ = 0.191 R_free_ = 0.201	R_factor_ = 0.188 R_free_ = 0.205	2WKJ	I04

*As defined in Nespolo *et al*.,^35^.

**THB refers to the competitive inhibitor (2 *R*,3 *R*)-2,3,4-trihydroxy-*N*,*N*-dipropylbutanamide, as reported in Campeotto *et al*.^16^. ***Refinement statistics were not of enough quality for model and data deposition, although data analysis was beneficial for the discussion presented here and the raw images were deposited in the public Zenodo database.

**Figure 2:**
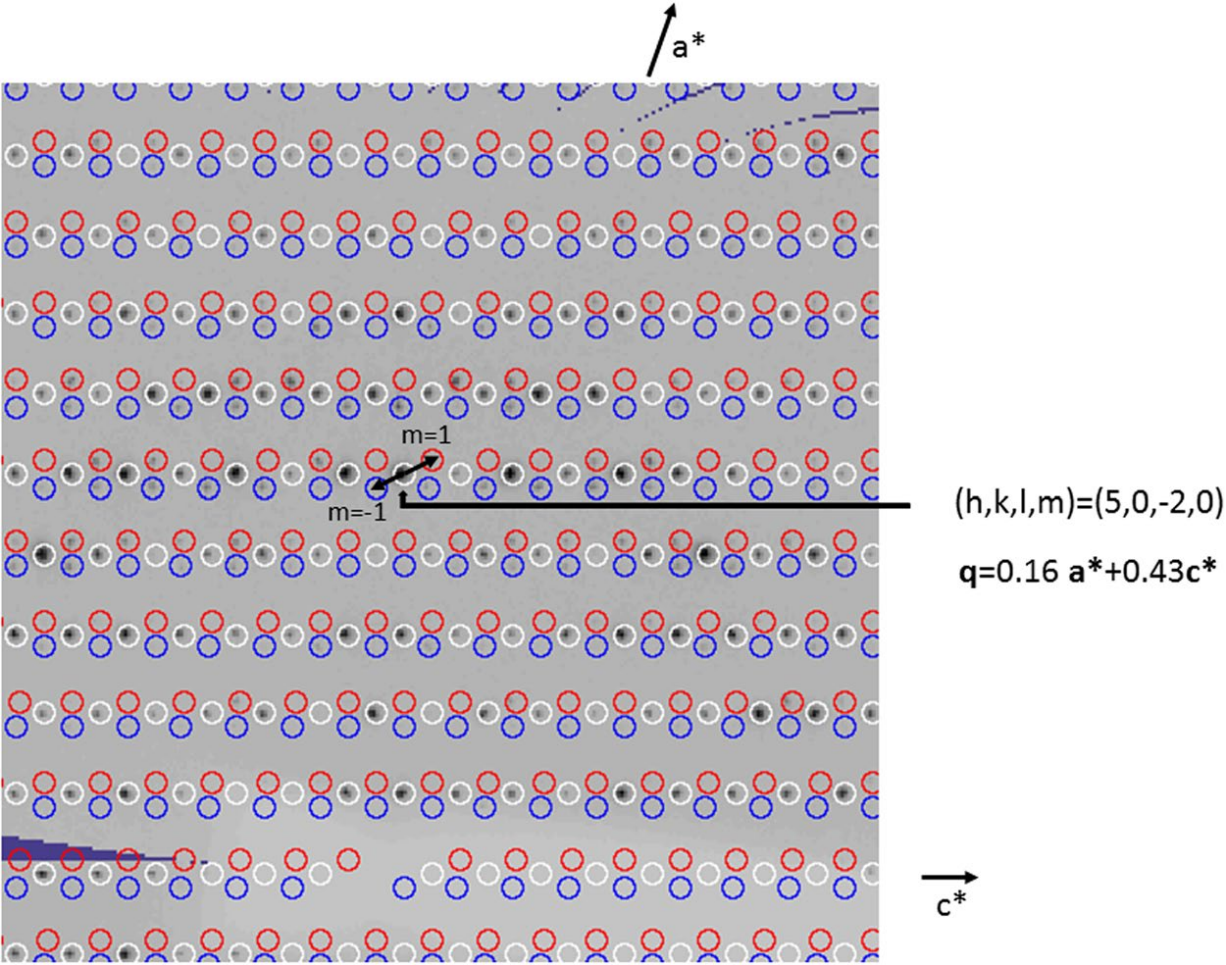
Precession reconstruction using of reciprocal space slice h0l of 2WNN (with *Precession* in the EVAL suite). The main lattice is coloured white. Satellite reflections with m = 1 and −1 are coloured red and blue, respectively. Satellites of (5, 0, −2) are indicated by arrows.

All the datasets in crystal form I were therefore indexed with Dirax^21^ to determine whether incommensurate modulation was present. This was indeed the case for four of the seven datasets, three of which were deposited in the PDB: 2WNN, 2WNQ, 2W05, whilst one, called Y137A, was not, due to unsatisfactory statistics. In those cases, reflections could be indexed and assigned either to the main lattice or to the satellite reflections with order m = −1 or 1 (see 2WNN as example in Fig. 2). No evidence of splitting of the main lattice was found, implying that the pseudo-merohedral twinned lattices almost exactly overlap. The data were processed with Eval^10^ and scaled with SADABS^22^ in 2 /m point group symmetry. The resulting statics are shown in Table 2.

**Table 2:** Analysis of the presence of crystal modulation in the structures belonging to crystal form I with EVAL15 package for modulated structures.

Dataset ID	2WNN	2WNQ	2WNZ	2WO5	2WPB	4BWL	Y137A
Bravais	P	P	P	P	P	P	P
Pointgroup	2/m	2/m	2/m	2/m	2/m	2/m	2/m
Cell axes a,b,c (Å)	54.8, 142.8, 83.7	54.8, 142.1, 84.5	57.4, 143.0, 83.9	54.6, 141.9, 84.0	56.8, 143.5, 84.2	56.1, 143.5, 83.6	54.8, 142.4, 83.7
alpha (Â°)	90.00, 109.0, 90.00	90.00, 108.9, 90.00	90.00, 109.9, 90.00	90.00, 108.9, 90.00	90.00, 109.8, 90.00	90.00, 109.6, 90.00	90.00, 109.0, 90.00
qvx1*	0.16	0.14	—	0.18	—	—	0.22
qvy1*	0.00	0.00	—	0.00	—	—	0.00
qvz1*	0.43	0.42	—	0.42	—	—	0.42
Resolution (Å)	41.9-1.65	49.9-1.80	38.7-1.80	48.7-2.20	47.8-2.05	49.6-1.80	49.9-1.80
Rmerge	0.070 (0.482)	0.081 (0.643)	0.059 (0.445)	0.121 (0.619)	0.084 (0.367)	0.088 (1.043)	0.099 (0.751)
Rmeas	0.083 (0.585)	0.096 (0.769)	0.069 (0.543)	0.144 (0.737)	0.099 (0.429)	0.103 (1.217)	0.119 (0.899)
Rpim	0.044 (0.327)	0.052 (0.417)	0.036 (0.307)	0.077 (0.395)	0.052 (0.221)	0.053 (0.623)	0.064 (0.489)
<I/sigI>	10.6 (1.8)	8.5 (1.5)	12.3 (1.8)	6.8 (2.1)	9.0 (2.9)	7.4 (1.0)	7.8 (1.4)
Completeness (%)	92.0 (61.2)	97.9 (97.0)	99.4 (94.6)	99.9 (99.7)	98.5 (97.9)	98.7 (98.0)	99.8 (98.5)
Redundancy	3.5 (3.2)	3.4 (3.3)	3.6 (3.1)	3.4 (3.4)	3.6 (3.7)	3.7 (3.8)	3.5 (3.4)
Reflections	1358205 (75799)	1111355 (107314)	413549 (32220)	625336 (60979)	282230 (28483)	419064 (42200)	1132807 (108078)
Unique	401726 (26603)	331525 (32667)	116035 (11021)	183588 (18294)	78040 (7722)	113330 (11261)	335150 (33038)
**Main reflections only**							
Rmerge	0.051 (0.340)	0.053 (0.412)	—	0.077 (0.362)	—	—	0.061 (0.503)
Rmeas	0.061 (0.413)	0.064 (0.492)	—	0.092 (0.432)	—	—	0.073 (0.603)
Rpim	0.033 (0.232)	0.035 (0.267)	—	0.050 (0.232)	—	—	0.039 (0.329)
<I/sigI>	12.6 (2.8)	11.7 (2.5)	—	8.1 (3.0)	—	—	8.5 (1.9)
Completeness (%)	91.9 (61.3)	97.2 (96.6)	—	99.8 (99.2)	—	—	99.7 (98.3)
Redundancy	3.5 (3.2)	3.3 (3.4)	—	3.4 (3.4)	—	—	3.4 (3.4)
No. Reflections	447223 (25340)	354109 (35713)	—	203706 (20152)	—	—	373048 (35922)

Four of the seven datasets were modulated. Statistics may differ slightly from Table 1 due to the processing being performed using a different package.

*q vector components.

All the modulated structures appeared also to be partially twinned (Tables 1 and 2). We speculate that the lack of modulation in 2WNZ and 4BWL is probably due to the larger unit cell axis a, which is large enough not to be incommensurate. With the P2_1_ indexing choice, POINTLESS initially assigned the space group C222_1_ but reflections belonging to one of the 2-fold axes were much stronger than the others (data not shown), which is consistent with pseudo-merohedral twinning in P2_1_, and indeed with this choice the structures could be easily solved.

However, in all the crystal forms, space group attribution was difficult or sometimes impossible and the choice of the point group was made based on the R_meas_ values^23^. Weak molecular replacement solutions could also be obtained in multiple space groups. As a general rule, whenever only a single lattice with no incommensurate modulation was present, indexing, data reduction and molecular replacement were possible, but the (non-twin) refinement stalled at R_factor_ and R_free_ values of 30–35% for all datasets (resolution range 1.45–2.3 Å, <*I*/σ(*I*)> cut off = 2.0) where we would expect R_factor_ values near or below 20% for well-behaved refinements.

### Twinning analysis

H and L twinning tests, as implemented in TRUNCATE^18^, were used as diagnostic tools for twinning. In our experience the L-test prediction was more consistent with estimates of twinning fraction performed internally in REFMAC.

**Figure 3:**
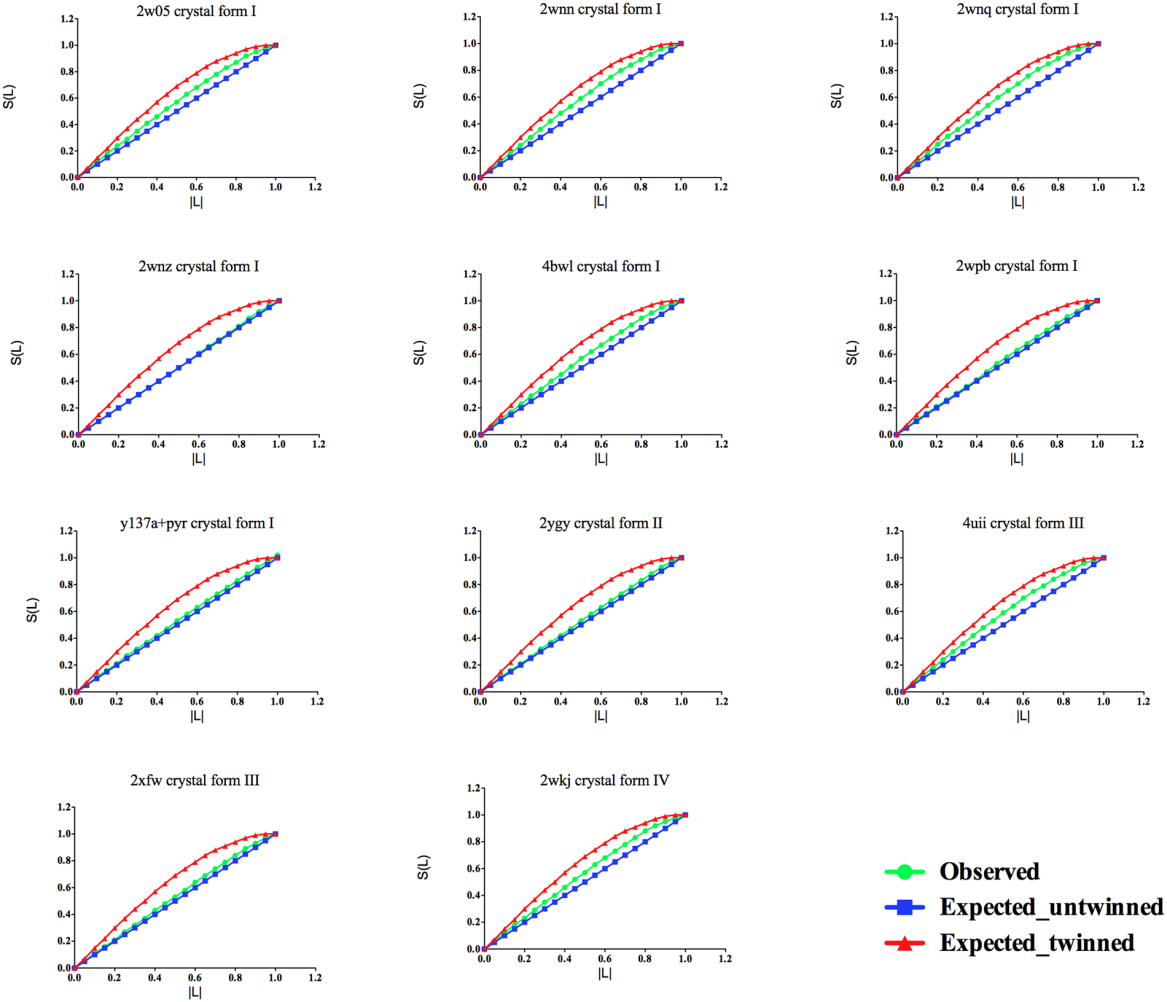
L-test analysis of the 11 NAL datasets reported here. For each crystal the crystal form is indicated. Cumulative intensity difference plot of the intensity difference of local pairs of intensities that are not twin-related |*L*| {*L* = [*I*(*h* 1) − *I*(*h* 2)]/[*I*(*h* 1) + *I*(*h* 2)]} against the cumulative probability distribution *N*(*L*) of the parameter *L*.

This is probably due to the fact that H and L tests are affected by experimental errors and lack discrimination power if one of the NCS-operation axes is parallel to twin-operation axis. However, the H-test requires for data to be merged in correct point group, and even then, in case of the NCS, it may seem to indicate partial twinning for data from single crystal. L-test is free from these two issues. For these reasons only the L-test is reported for the presented datasets (Fig. 3).

Micro-seeding techniques were employed in an attempt to avoid twinning by growing larger single crystals^24^. However, twinning persisted, suggesting that it was likely to be a nucleation phenomenon, which was perpetuated when twinned seed crystals were used as nuclei. Diffraction data were collected at 100 K following flash cooling of crystals in cryo-protectants, which could have been a source of lattice disorder. Data collection at room temperature from multiple crystals, however, also showed both split diffraction and significant twinning (data not shown), indicating that the disorder pre-existed in the crystals. Ligand soaking experiments were similarly excluded as a cause of the twinning.

**Figure 4:**
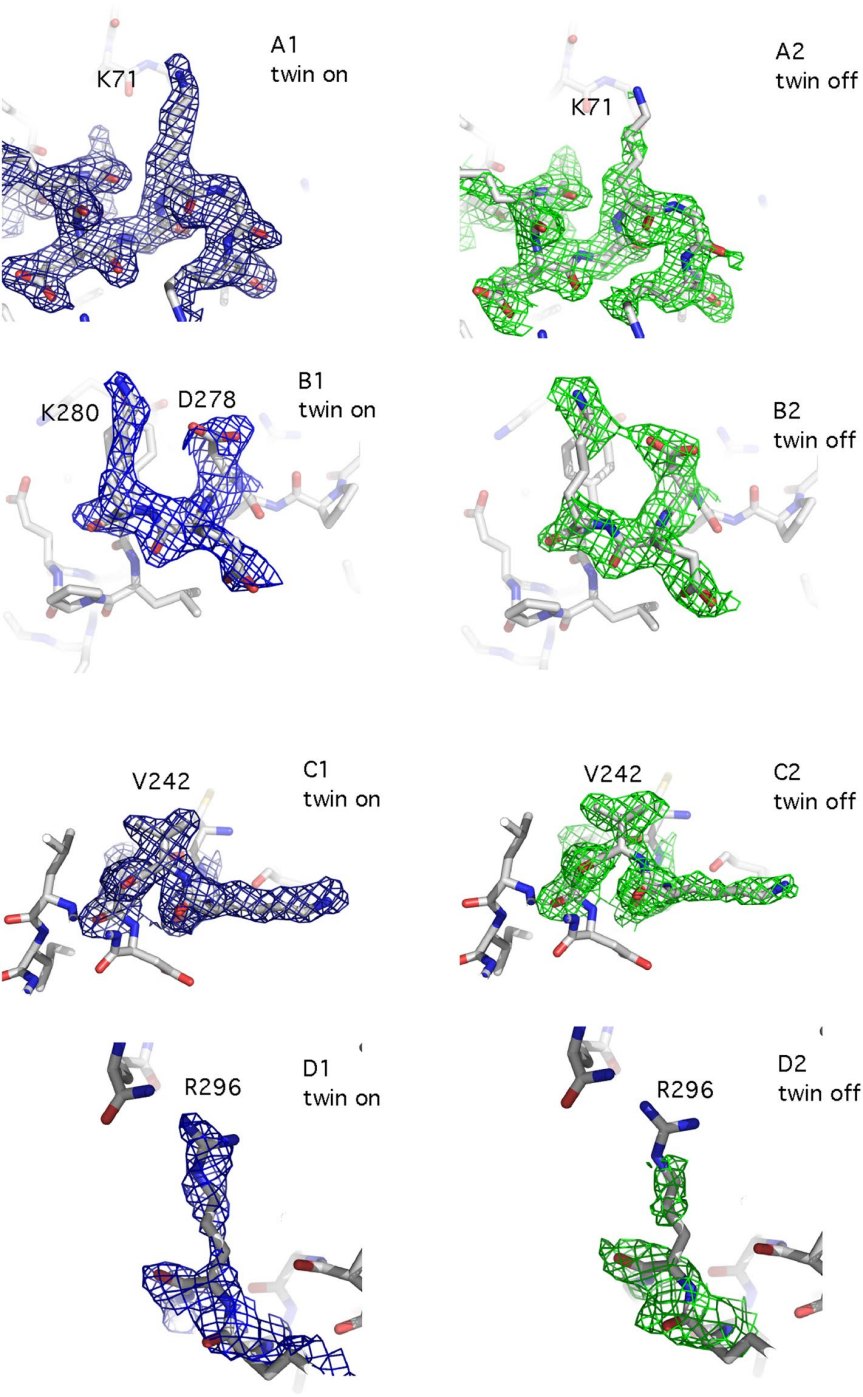
Comparison between equivalent portions of the electron density map before (A1, B1, C1, D1) and after (A2, B2, C2, D2) applying the twin option in REFMAC. The electron density maps refer to different regions of dataset 4BWL, which belongs to crystal form I and showed a twin fraction of almost 50%.

Refinement with the program REFMAC (versions 5.6 and 5.7) identified the twinning operator (−h, −k, h + l) for all the cases, in which twinning was detected. Twin refinement resulted in improved models with R_factor_ and R_free_ values ~18–20% (Table S2, data collection and final refinement statistics are summarised in Table 1). This improvement of the R_factor_ quality indices was accompanied by local improvements of the electron density maps, which became better defined and showed increased connectivity (Fig. 4). The best refined model for each crystal form was validated using ZANUDA^18^, which confirmed the space group assignment in all cases by transforming the individual space group into the lower symmetry space groups, followed by refinement of the corresponding models using REFMAC and selection of the model with highest symmetry from the ones with best refinement statistics.

### Crystal packing analysis

**Figure 5:**
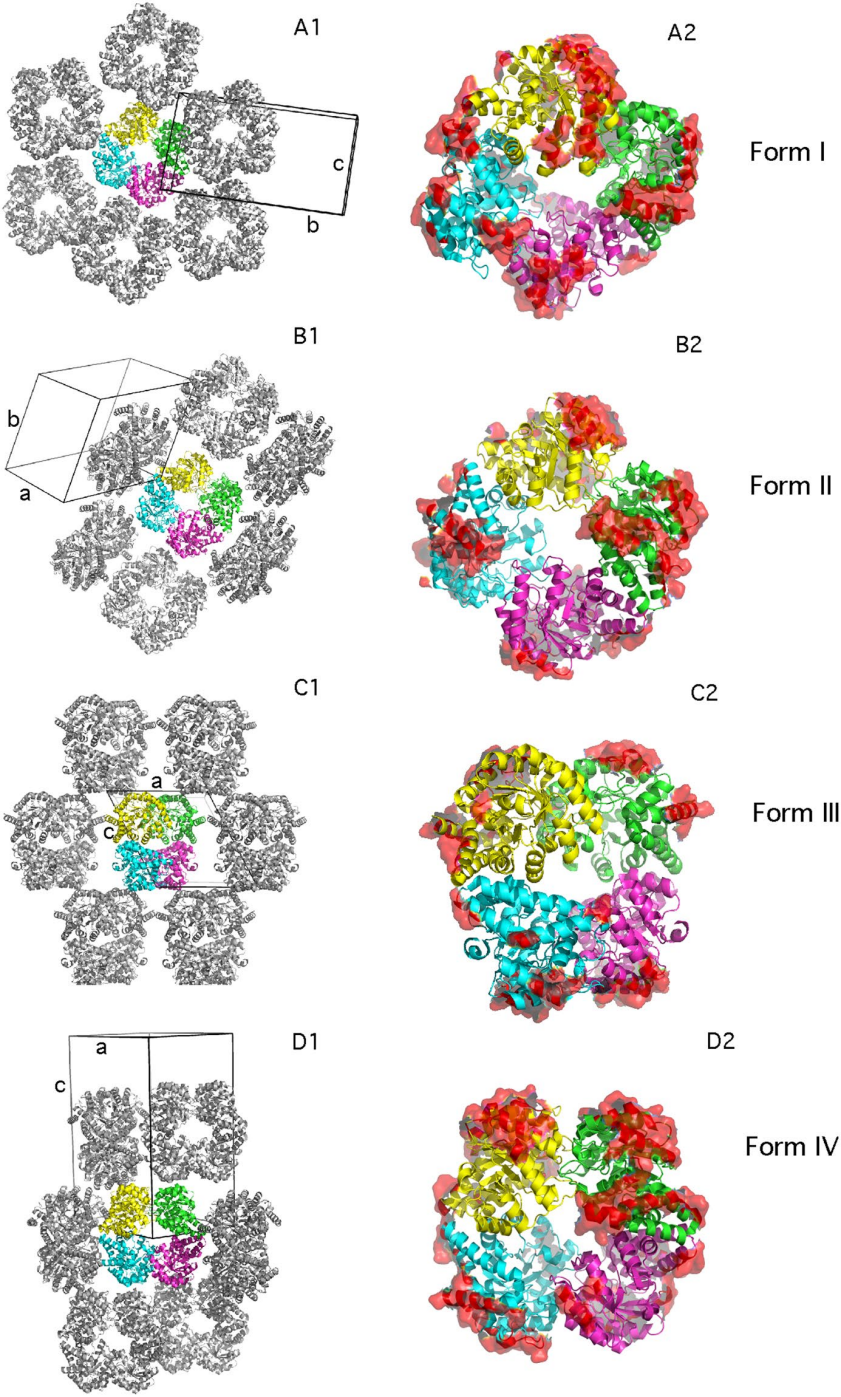
Crystal packing and crystal contacts in the four crystal forms of NAL. For each crystal form the crystal packing was inspected manually and the least overlapping orientation is presented as 2D layer (A1, B1, C1 and D1). The crystal contacts between tetramers in the given orientation are represented in more detail (A2, B2, C2, D2) as a red surface with the orientations of the tetramers kept the same as in the corresponding 2D layer. As examples of crystal forms I, II, III and IV, the structures of PDB code 2WNN, 2YGY, 2XFW and 2WKJ are represented respectively. Images were produced in PYMOL version 1.6.0.0.

Zanuda was used to expand the final refined models into space group P1 in order to compare packing in the different crystal forms. Inspection of the packing using the molecular graphics program COOT^25^ highlighted how not only the inter-monomer contacts within the NAL tetramers were different, but also the inter-tetramer contacts in the crystal lattice (Fig. 5). We speculate that the likelihood of NAL of crystallising in any one of the four forms is determined by small differences in the interfaces between tetramer during nucleation and the early stages of crystal growth. This process is kinetically and thermodynamically difficult to control and attempts to select for a specific crystal form were hindered by the fact that all four forms were obtained in the same crystallisation drops and therefore from identical crystallisation conditions. Surface accessible areas and free energies of interaction were calculated using PISA (Table 2)^26^. These did not show any significant differences in the strength of intra-tetramer interactions between the different crystal forms, consistent with our observation that all four crystal forms appeared in the same crystallisation drops.

### Future developments

The presented datasets were the result of an extensive screening at the data collection stage and of an extensive processing at the data reduction and data refinement stages with very low success rate (Fig. S3). The development of twin refinement in REFMAC, which at the time was only implemented in the experimental version of the program, allowed the determination of several apo- and ligand bound structures of NAL and the proposal of the first detailed mechanism of the enzyme reaction^15,16^. Although twin refinement is currently included in REFMAC, the presented datasets are still a challenging test for current indexing and scaling programs, including iMosflm, LABELIT^27^ and XDS^28^, and they therefore offer an excellent opportunity for the development of these softwares.

Several improvements in MX software are still very desirable in the part of dealing with pathological data. This includes robust diagnostics and warning messages, automated space group assignment in at least obvious cases of twinning, and, importantly, robust integration of partially overlapping reflections and communication of all the necessary data and metadata to a refinement program. Crystal modulation was also detected only after structure deposition and although this had no effect on data processing in the presented cases, its diagnosis should be implemented to avoid reflection overlaps, which in severe cases can seriously hamper indexing, data reduction and ultimately phasing and satisfactory refinement.

## Methods

### Data collection and structure solution

We have previously reported several structures of wild type NAL and engineered variants and NAL crystals were obtained as previously described^15,16^. NAL crystals are plate-shaped and tend to grow in clusters and therefore micro-seeding experiments were required to obtain single large crystals. Crystal cryo-protection was achieved by serial transfer of the crystals through mother liquor containing 20% and then 25% v/v PEG 400, with 2 minutes soak time at each step. Eleven datasets were collected from single crystals at Diamond Light Source (beamlines I02, I03 and I04), at 100 K with a 1 s exposure and an oscillation of 0.5° per image and using a Q315 ADSC CCD detector. Data were processed using iMOSFLM and scaled and merged using SCALA^29^.

In the case of the datasets of crystal form I, diffraction patterns were inspected with DIALS^20^ for the presence of satellite reflection, indexed with Dirax^22^ and processed with EVAL15^10^. Scaling was performed with SADABS in 2/m point group symmetry. The results are shown in Table 2. For structure refinement only the main lattice reflections from MOSFLM were used, ignoring the weak satellite reflections.

In each dataset five percent of the reflections were excluded from the refinement and constituted the R_free_ set. A new R_free_ set was generated randomly for each new crystal form and then transferred to all datasets belonging to the same crystal form.

The first crystal structure obtained for each crystal form was solved by molecular replacement using PHASER^30^ and 1NAL as a starting model^31^, while refinement against other datasets of the same crystal form started with 20 cycles of rigid body refinement (resolution range 10.0–6.0 Å) followed by 10 cycles of preliminary restrained refinement (whole resolution range) in REFMAC5.

### Refinement and Crystal packing analysis

Refinement was performed using REFMAC 5.6 or 5.7 (i.e. the latest version at the time of deposition or final refinement for each structure), with and without twin refinement, both for electron density calculations and evaluation of statistics. Refinement was performed with the same settings for all reported structures, *i.e*. 20 cycles per run (using the whole resolution range of the data), a weight matrix of 0.1^32^, with riding hydrogen atoms.

For all structures involved, regardless of whether the unit cell parameters allowed for twinning by merohedry or not, the refinement protocol was identical and included twin-refinement in the final refinement rounds^32^. If no twinning operations are present, the twin refinement option means that REFMAC uses approximation to the likelihood target rather than its exact version. Such usage therefore only makes sense for comparison of refinement results for twinned and untwined crystals.

R_factor_ and R_free_ values were compared before and after twin refinement.

The values of the obliquity angle, which are a measure of pseudo-symmetry, were monitored and manual inspection of the diffraction pattern were performed with ADXV^33^.

The concept of obliquity is a measure of the overlap of lattices on the individuals forming a twin and Friedel provided a formal mathematical description since the early day of crystallography^34^. Briefly, the closest is the obliquity angle to zero, the more likely is the presence of merohedral twinning^35^, as the two twin lattices tend to overlap. Values of obliquity close to zero are, however, only a possible indicator that twinning may be present but not a fixed rule, as some of the presented datasets highlight. For instance, in the case of crystal form I and III, the obliquity angle is small enough to allow twinning in some cases, whilst in crystal form II is too large for twinning to occur (Table 1).

Manual model building was performed in COOT. Zanuda was used to expand the unit cell of each crystal form into P1 for each crystal form and these were refined against the data processed in P1 in order to confirm the correctness of the space group assignment in each case.

In order to assess how the four crystal forms of NAL were related to each other, Csymmatch from CCP4 was used to bring all the P1-expanded structures to the same origin and NCONTACT^18^ was used to calculate inter-tetramer contacts. The input file from NCONTACT was used in PYMOL to visualise the contact surface between monomers. Surface accessibility areas and crystal contact energy were calculated using PISA^26^ (Table S3).

## Electronic supplementary material

Supplementary information

## Data Availability

The datasets (raw diffraction images) discussed in this manuscript have been deposited in the publicly available database zenodo at, https://doi.org/ 10.5281/zenodo.54568 and 10.5281/zenodo.1240503. Structural models and processed structure factor data deposited in the PDB are available under the accession codes given in Table 1, with the exception of dataset Y137A, as the R factor indices were not satisfactory for PDB deposition.
