# Supplementary material for: Pathological macromolecular crystallographic data affected by twinning, partial-disorder and exhibiting multiple lattices for testing of data processing and refinement tools
Source: Sci Rep. 2018 Oct 5;8:14876. doi: 10.1038/s41598-018-32962-6 (PMC6173773; doi:10.1038/s41598-018-32962-6)
Supplement: Supplementary file 1 — Supplementary information [file 41598_2018_32962_MOESM1_ESM.docx]

**Supplementary information related to manuscript:**

“Pathological macromolecular crystallographic data affected by twinning, partial-disorder and exhibiting multiple lattices for testing of data processing and refinement tools”

### Authors

Ivan Campeotto, Andrey Lebedev, Antoine M.M. Schreurs, Loes M.J. Kroon-Batenburg, Edward Lowe, Simon E.V. Phillips, Garib N. Murshudov and Arwen R. Pearson

**Figure S1**


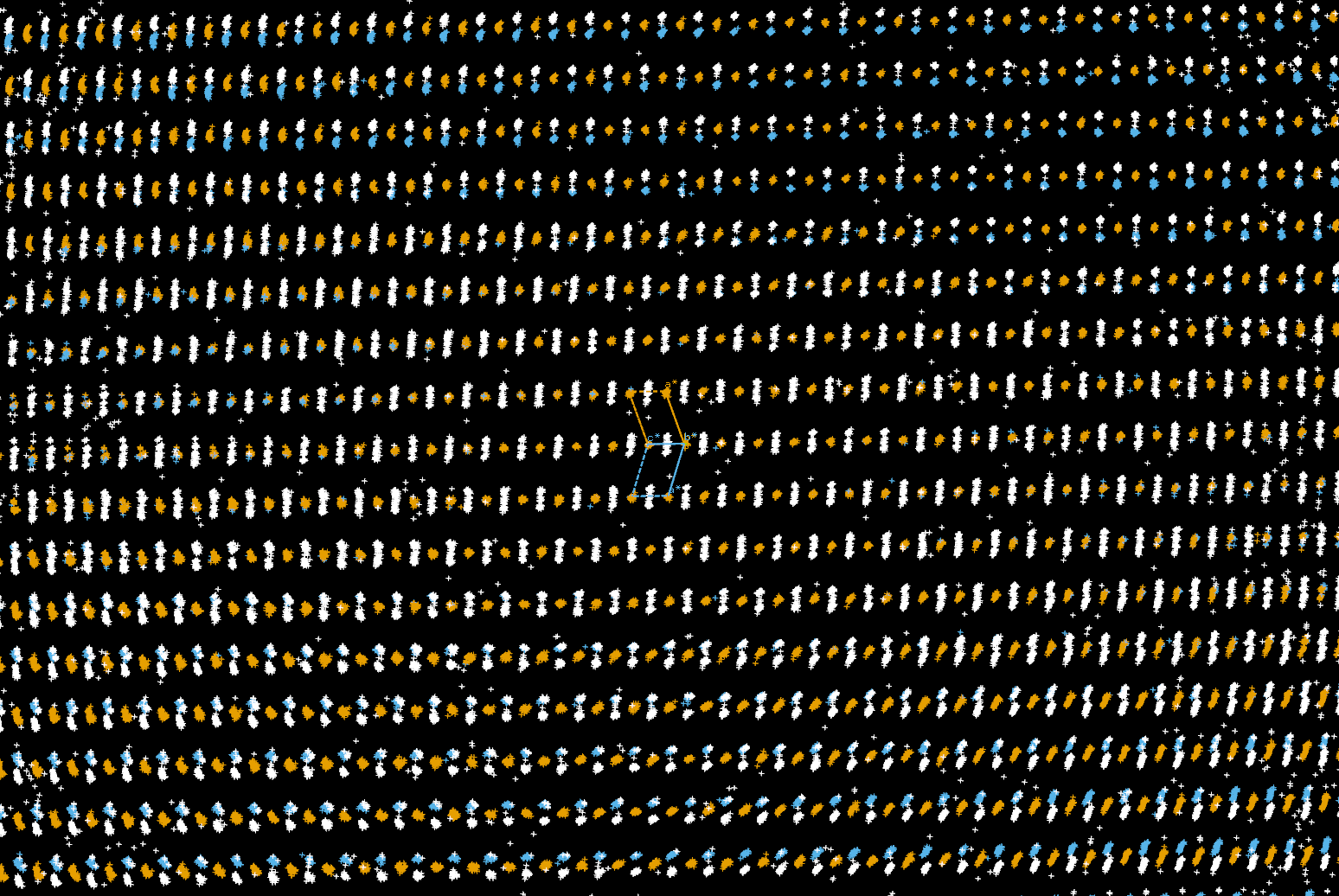


**Figure S2**


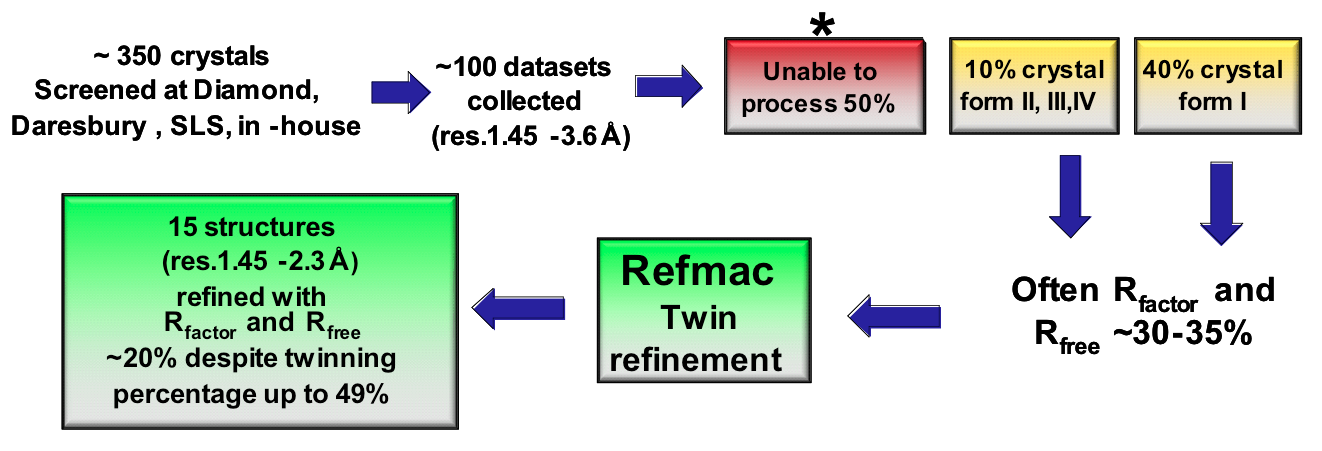


**Figure S3**

### Table S1 Different crystal forms of the enzyme NAL.

| Crystal form | Unit cell parameters(Å,°) | Space group |
| --- | --- | --- |
| I | a = 55, b = 142, c = 84  α = 90, β = 109, γ = 90 | *P*2_1_ |
| II | a = 84, b = 95, c = 91  α = 90, β = 116, γ = 90 | *P*2_1_ |
| III | a = 78, b = 108, c = 148  α = 90, β = 116, γ = 90 | *P*2_1_ |
| IV | a =78, b = 116, c = 84  α = β = γ = 90 | *P*2_1_2_1_2_1_ |

**Table S2 Crystallographic data collection and refinement statistics. ^a^ Two additional molecules of non-covalently bound pyruvate are observed in subunits C and D.**

| Parameters | Wild-type | Wild-type+pyr | E192N | E192N+pyr | E192N+pyr+THB* |
| --- | --- | --- | --- | --- | --- |
| Diamond beamline | I02 | I03 | I04 | I03 | I03 |
| Space group | P2_1_ | P2_1_ | P2_1_ | P2_1_ | P2_1_ |
| Crystal form | I | I | I | I | I |
| *a*, *b*, *c* (Å), (^o^) | 54.8, 142.2, 84.2, 109.0 | 54.7, 142.5, 83.6, 109.2 | 54.6, 142.8, 84.5, 109.0 | 56.9, 143.0, 83.9, 109.8 | 57.0, 143.7, 83.3, 109.9 |
| Resolution (Å) | 79.56-2.20 (2.26-2.20) | 79.1-1.65 (1.74-1.65) | 47.60-1.80 (1.90-1.80) | 79.06-1.80 (1.95-1.80) | 79.31-2.05 (2.16-2.05) |
| *R*_merge_ | 0.102 (0.365) | 0.060 (0.317) | 0.086 (0.388) | 0.070 (0.430) | 0.09 (0.438) |
| *R*_pim_ (all I^+^ & I^-^) | 0.063 (0.219) | 0.038 (0.215) | 0.054 (0.033) | 0.043 (0.269) | 0.056 (0.273) |
| *<I>*/sd <I> | 8.5 (3.4) | 13.5 (3.2) | 8.3 (2.8) | .0 (2.5) | 8.6 (2.6) |
| Completeness (%) | 99.9 (100) | 91.2 (60.8) | 98. 4 (97.2) | 100 (99.9) | 98.6 (96.3) |
| Redundancy | 3.7 (3.7) | 3.4 (2.9) | 3.6 (3.5) | 3.6 (3.5) | 3.5 (3.4) |
| Wilson B factor (Å^2^) | 34.8 | 31.7 | 26.8 | 21.8 | 24.5 |
| No. reflections | 197621 (29177) | 449770 (37158) | 398910 (55837) | 391185 (54278) | 277866 (37606) |
| No. unique | 54006 (7847) | 131694 (12746) | 110985 (15919) | 107356 (15671) | 78597 (11141) |
| Resolution (Å) | 79.56-2.20 (2.26-2.20) | 79.1-1.65 (1.69-1.65) | 47.60-1.80 (1.90-1.80) | 79.06-1.80 (1.95-1.80) | 79.31-2.05 (2.16-2.05) |
| *R*_factor_ | 0.211 (0.259) | 0.209 (0.261) | 0.197 (0.261) | 0.188 (0.301) | 0.190 (0.281) |
| *R*_free_ | 0.271 (0.356) | 0.247 (0.344) | 0.253 (0.391) | 0.225 (0.344) | 0.234 (0.337) |
| Twinning fraction | 0.338 | 0.334 | 0.463 | none | 0.06 |
| Twinning operator | -h, -k, h+l | -h, -k, h+l | -h, -k, h+l | none | -h, -k, h+l |
| No. atoms |  |  |  |  |  |
| Protein | 9166 | 9166 | 9178 | 9292 | 9246 |
| Water | 169 | 758 | 316 | 654 | 540 |
| Ligands | - | 31 (PEG400, Na^+^) | 4 (Cl^-^) | 24 (PEG400, pyruvate^a^) | 60 (THB) |
| Average B-factors (Ǻ^2^) |  |  |  |  |  |
| Protein | 32.9 | 22.8 | 28.0 | 22.8 | 28.4 |
| Waters | 27.9 | 26.7 | 26.7 | 27.2 | 31.9 |
| Ligands | - | 32.4 | 26.3 | 45.8 | 50.1 |
| R.m.s deviations |  |  |  |  |  |
| Bond lengths (Å) | 0.013 | 0.012 | 0.011 | 0.009 | 0.012 |
| Bond angles (º) | 1.46 | 1.80 | 1.33 | 1.14 | 1.28 |
| Ramachandran most favoured (%) | 100 | 100 | 100 | 100 | 100 |
| PDB code | 2WO5 | 2WNN | 2WNQ | 2WNZ | 2WPB |

* THB = (2R,3R)-2,3,4-trihydroxy-N,N-dipropylbutanamide

**Table S2 continued Crystallographic data collection and refinement statistics.**

| Parameters | Y137A + pyr | Wild-type | E192N+pyr | E192N/Y137F+pyr | Y137A+Neu5Ac | E192N+pyr |
| --- | --- | --- | --- | --- | --- | --- |
| Beamline | I04 | I02 | I02 | I02 | I02 | I04 |
| Space group | *P*2_1_ | P2_1_ | *P*2_1_ | *P*2_1_ | *P*2_1_ | *P*2_1_2_1_2_1_ |
| Crystal form | I | I | II | III | III | IV |
| *a*, *b*, *c* (Å), *β*(^o^) | 54.6 142.2 83.6, 109.0 | 56.0, 143.2, 83.4, 109.5 | 84.3 95.9 91.4, 115.3 | 78.1 116.5 83.7, 117.9 | 77.9 116.7 83.7, 118.1 | 78.3 108.5 148.3 |
| Resolution (Å) | 48.54-1.80 (1.90-1.80) | 49.50-2.0 (2.11-2.0) | 47.94-1.90 (2.00-1.90) | 37.03-1.65 (1.74-1.65) | 73.92-1.80 (1.90-1.80) | 63.41-1.45 (1.53-1.45) |
| *R*_merge_ | 0.08 (0.547) | 0.09 (0.41) | 0.102 (0.324) | 0.087 (0.444) | 0.122 (0.534) | 0.077 (0.375) |
| *R*_pim_ (all I^+^ & I^-^) | 0.049 (0.336) | 0.057 (0.254) | 0.069 (0.203) | 0.102 (0.521) | 0.131 (0.576) | 0.036 (0.172) |
| *<I>*/sd <I> | 10.8 (2.7) | 9.6 (2.8) | 7.0 (3.7) | 10.9 (2.8) | 13.4 (4.9) | 11.6 (3.4) |
| Completeness (%) | 99.9 (100.0) |  | 99.4 (99.5) | 100 (99.9) | 100 (100) | 96.2 (89.5) |
| Redundancy | 3.7 (3.7) | 3.6 (3.5) | 3.1 (3.1) | 3.7 (3.7) | 7.2 (7.3) | 5.1 (4.9) |
| No. reflections | 409173 (60138) | 293838 (40147) | 318719 (46597) | 584978 (85524) | 884843 (130078) | 1095072 (138933) |
| No. unique | 111282 (16303) | 82202 (11558) | 102699 (15024) | 158593 (23132) | 122053 (17798) | 213226 (28642) |
| Resolution (Å) | 48.54-1.80 (1.90-1.80) |  | 47.94-1.90 (2.00-1.90) | 37.03-1.65 (1.74-1.65) | 73.92-1.80 (1.90-1.80) | 63.41-1.45 (1.53-1.45) |
| *R*_factor_ | 0.275 (0.292) | 0.179 (0.306) | 0.209 (0.300) | 0.165 (0.220) | 0.205 (0.240) | 0.167 (0.280) |
| *R*_free_ | 0.329 (0.386) | 0.235 (0.379) | 0.249 (0.340) | 0.186 (0.230) | 0.235 (0.280) | 0.189 (0.290) |
| Twinning fraction | 0.149 | 0.488 | none | 0.096 | 0.328 | none |
| Twinning operator | -h, -k, h+l | -h, -k, h+l | - | -h –k h+l | -h, -k, h+l | - |
| No. atoms |  |  |  |  |  |  |
| Protein | 9173 | 9149 | 9260 | 9389 | 9381 | 9336 |
| Water | 106 | 102 | 653 | 977 | 745 | 1307 |
| Ligands | 20 | 97 | - | 87 | 61 | 34 |
| Average B-factors (Ǻ^2^) |  |  |  |  |  |  |
| Protein | 28.2 | 28.2 | 19.8 | 15.4 | 15.3 | 14.4 |
| Waters | 29.0 | 19.7 | 25.8 | 22.3 | 20.5 | 16.5 |
| Covalently bound pyruvate | 23.5 | 26.4 | - | 9.5 | 14.2 | 10.8 |
| ManNAc | - | 27.6 | - | - | - | - |
| Neu5Ac | - | 25.4 | - | - | - |  |
| Noncovalently bound, pyr. | - | - | - | 31.9 | 32.9 | 28.7 |
| PEG 400 | - | 35.5 | - | 32.8 | - | 50.0 |
| R.m.s deviations |  |  |  |  |  |  |
| Bond lengths (Å) | 0.020 | 0.012 | 0.009 | 0.006 | 0.008 | 0.012 |
| Bond angles (º) | 2.27 | 1.34 | 1.17 | 1.05 | 1.14 | 1.346 |
| Ramachandran most fav.(%) | 100 | 100 | 100 | 100 | 100 | 100 |
| PDB code | n./a. | 4BWL | 2YGY | 2XFW | 2YGZ | 2WKJ |

**Table S3 Interface interaction intra- and inter-tetramer as calculated**

**by PISA for crystal form I of NAL (PDB code: 2WNN).**

| Interface | Type | Symmetry operation | Surface  (Ǻ^2^_)_ | ΔG  (Kcal/mol) |
| --- | --- | --- | --- | --- |
| **D----A** | Intra dimer | x,y,z | 1202.5 | -18.6 |
| **C----B** | Intra dimer | x,y,z | 1188.2 | -18.3 |
| **D----A** | Inter dimer | x,y,z | 1013.5 | -10.2 |
| **C----A** | Inter dimer | x,y,z | 1004.4 | -10.8 |
| **C----D** | Inter dimer | x,y,z-1 | 323.3 | 0.1 |
| **C----D** | Inter dimer | x-1,y,z-1 | 285.4 | -0.2 |
| **B----A** | Inter dimer | -x+1,y-1/2,z+1 |  |  |

**Figure Legends**

**Figure S1 Crystal gallery of NAL and NAL variants.** Different crystal morphologies were observed in the same crystallisation conditions and often within the same crystallisation drop. It was not possible to find a correlation between crystal shape and one of the four crystal forms. Crystals are not shown on the same scale; the average size of the crystals was ~300 µm.

**Figure S2** DIALS reciprocal lattice viewer showing both indexed lattices in orange and in blue, whilst unindexed reflections are coloured in white.

**Figure S3** General statistics of the project.*Inability to process these datasets was due to problems at the autoindexing step or in data merging due to poor or split diffraction. Eleven of the fifteen final structures are presented in the current manuscript.
